# Inferring Tunicate Relationships and the Evolution of the Tunicate Hox Cluster with the Genome of *Corella inflata*

**DOI:** 10.1093/gbe/evaa060

**Published:** 2020-03-25

**Authors:** Melissa B DeBiasse, William N Colgan, Lincoln Harris, Bradley Davidson, Joseph F Ryan

**Affiliations:** e1 Whitney Laboratory for Marine Bioscience, University of Florida; e2 Department of Biology, University of Florida, Gainesville; e3 Department of Biology, Swarthmore College, Swarthmore, Pennsylvania

**Keywords:** compositional heterogeneity, Enterogona, gene loss, PacBio, Phlebobranchia, phylogenomics

## Abstract

Tunicates, the closest living relatives of vertebrates, have served as a foundational model of early embryonic development for decades. Comparative studies of tunicate phylogeny and genome evolution provide a critical framework for analyzing chordate diversification and the emergence of vertebrates. Toward this goal, we sequenced the genome of *Corella inflata* (Ascidiacea, Phlebobranchia), so named for the capacity to brood self-fertilized embryos in a modified, “inflated” atrial chamber. Combining the new genome sequence for *Co. inflata* with publicly available tunicate data, we estimated a tunicate species phylogeny, reconstructed the ancestral Hox gene cluster at important nodes in the tunicate tree, and compared patterns of gene loss between *Co. inflata* and *Ciona robusta*, the prevailing tunicate model species. Our maximum-likelihood and Bayesian trees estimated from a concatenated 210-gene matrix were largely concordant and showed that Aplousobranchia was nested within a paraphyletic Phlebobranchia. We demonstrated that this relationship is not an artifact due to compositional heterogeneity, as had been suggested by previous studies. In addition, within Thaliacea, we recovered Doliolida as sister to the clade containing Salpida and Pyrosomatida. The *Co. inflata* genome provides increased resolution of the ancestral Hox clusters of key tunicate nodes, therefore expanding our understanding of the evolution of this cluster and its potential impact on tunicate morphological diversity. Our analyses of other gene families revealed that several cardiovascular associated genes (e.g., *BMP10*, *SCL2A12*, and *PDE2a*) absent from *Ci. robusta,* are present in *Co. inflata*. Taken together, our results help clarify tunicate relationships and the genomic content of key ancestral nodes within this phylogeny, providing critical insights into tunicate evolution.

## Introduction

Extensive research on tunicates has contributed substantial insights into the mechanisms and evolution of early embryonic development. Because they are the closest living relative of vertebrates, comparative studies of tunicate genomes can provide unique insights into vertebrate origins and subsequent genomic changes underlying vertebrate diversification (Delsuc et al. 2006). Furthermore, tunicates are a highly diverse clade with an extraordinary range of life history traits and high regenerative potential, making them ideal for examining a range of questions including the evolution of sexual versus asexual reproduction, colonial versus solitary life strategies, and the evolution of regenerative processes (Lemaire 2011; Kassmer et al. 2019). Tunicates are also of interest economically given some species are invasive pests (Lambert 2007) and others are potential food and biofuel sources (Lambert et al. 2016). Tunicates exhibit a remarkably high rate of genome evolution while maintaining a stringently conserved developmental program (Berná and Alvarez-Valin 2014). Thus, comparative studies of tunicate genomes represent an ideal platform for examining how constraints guide the evolution of developmental genes and the regulatory connections between them (Stolfi et al. 2014).

Tunicate phylogenetic relationships remain poorly resolved across taxonomic levels. The approximately 3,000 species have historically been divided into three classes: Ascidiacea (sea squirts), Thaliacea (pelagic salps, doliolids, pyrosomes), and Appendicularia (larvaceans) (Berrill 1936). After Sorberacea (deep water, “ascidian-like”) was shown to be closely related to molgulid ascidians rather than a stand-alone class (Tatián et al. 2011) and ribosomal and mitochondrial phylogenies revealed that Ascidiacea was paraphyletic (Swalla et al. 2000; Zeng and Swalla 2005; Singh et al. 2009; Tsagkogeorga et al. 2009; Rubinstein et al. 2013), the following three clades were proposed: 1) Stolidobranchia, 2) Appendicularia, and 3) Phlebobranchia + Aplousobranchia + Thaliacea. The relationships within these clades, however, have remained unresolved. For example, phylogenies based on 18S and morphological traits conflicted in the placement of salps, pyrosomes, and doliolids within Thaliacea (Tsagkogeorga et al. 2009; Govindarajan et al. 2011; Braun et al. 2020). Three phylogenomic studies (Alié et al. 2018; Delsuc et al. 2018; Kocot et al. 2018) were congruent with one important exception regarding the Phlebobranchia, a group that includes *Ciona robusta*, formerly *Ciona intestinalis* type A, hereafter *Ci. robusta*, and *Corella inflata*, hereafter *Co. inflata* (Stolfi et al. 2015). Kocot et al. (2018) reported Aplousobranchia was sister to a monophyletic Phlebobranchia, whereas Delsuc et al. (2018) found Phlebobranchia was not monophyletic, as *Ci. robusta* was sister to a clade that included Aplousobranchia and the rest of Phlebobranchia (Alié et al. [2018] did not include Aplousobranchia in their analysis). None of these phylogenomic studies included representatives from all of the three major Thaliacea lineages (i.e., Doliolida, Salpida, and Pyrosomatida).

Phylogenetic relationships within tunicate genera are also complex. For example, *Ci. robusta*, a shallow water species common in harbors and semienclosed basins, was historically thought to have a cosmopolitan distribution, although evidence of variation in morphology (Caputi et al. 2007; Pennati et al. 2015), physiological tolerance (Dybern 1967; Renborg et al. 2014), and reproductive compatibility among populations existed (Suzuki et al. 2005; Caputi et al. 2007; Sato et al. 2014). Understanding species boundaries in *Ci. robusta* is critical given that this species has been the foundation for decades of developmental research (Satoh and Jeffery 1995; Satoh et al. 2003) and its genome was published in 2002 (Dehal 2002). Recently, two genetically divergent and largely geographically isolated forms, *Ci. robusta* and *Ci. intestinalis* (formerly *Ci. intestinalis*, type B as described by Millar [1953]) have been designated as distinct species using molecular and morphological methods (Brunetti et al. 2015).

Past tunicate studies have made considerable contributions to our understanding of developmental processes in two phlebobranchs, *Ci. robusta* and *Phallusia mammillata* (Zalokar and Sardet 1984; Glardon et al. 1997; Passamaneck and Di Gregorio 2005; Davidson 2007; Roure et al. 2014), along with a limited set of stolidobranchs: 1) *Halocynthia roretzi* (Wada et al. 1995; Hirano and Nishida 2000), 2) a set of three molgulid species (Huber et al. 2000; Stolfi et al. 2014; Racioppi et al. 2017) and 3) the colonial tunicate *Botryllus schlosseri* (Kassmer et al. 2016; Manni et al. 2019). More recently, substantial progress has been made in exploring the development of the appendicularian, *Oikopleura dioica* (Seo 2001; Ganot and Thompson 2002; Cañestro et al. 2005; Wang et al. 2015). In particular, genome data (Seo et al. 2004; Naville et al. 2019) have led to a better understanding of the evolution of the tunicate Hox cluster, an array of homeobox-containing genes that are key developmental genes involved in specifying the primary body axis of most animals (McGinnis and Krumlauf 1992). Most studies of tunicate Hox genes to date have emphasized the breakup of the tunicate cluster despite partial conservation of colinear expression patterns (e.g., Ikuta et al. 2004).

**Fig. 1.**
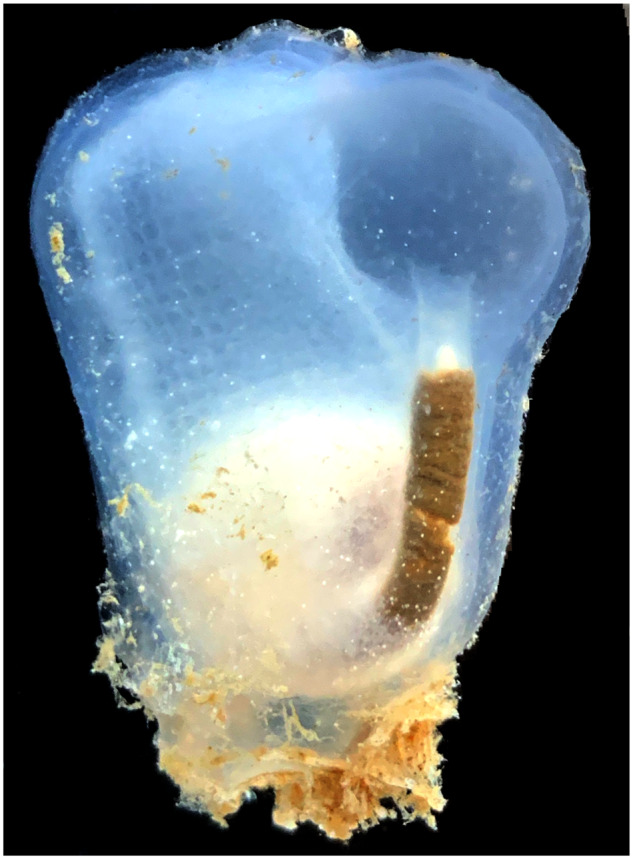
—*Corella inflata*. Photograph of the tunicate *Co. inflata* originally described by A. G. Huntsman in 1912 at Vancouver Island. Photo of a specimen collected from Friday Harbor, WA by B. Davidson.

Data from additional tunicate species are necessary to reliably reconstruct the evolution and diversification of tunicate and vertebrate clades from their last common ancestor. The first steps toward establishing new tunicate models include generating annotated genomes and a robust tunicate phylogeny. Toward this goal, we present the genome and transcriptome of *Co. inflata* (Ascidiacea, Phlebobranchia, fig. 1), a comparative tunicate genome analysis, and a revised tunicate tree of life combining data generated here for *Co. inflata* with previously published transcriptome data.

*Corella inflata* represents an attractive new model. Comparative analysis of the *Co. inflata* genome will help reconstruct the genome architecture of key ancestral tunicate nodes. Specifically, comparisons with *Ci. robusta* will help to delineate how well this primary tunicate model organism represents tunicate genomes in general. Additionally, established protocols exist for transgenesis of *Co. inflata* embryos, permitting stringent cross-species analyses of developmental gene network evolution (Colgan et al. 2019).

Although many ascidians are self-infertile hermaphrodites that breed through free spawning, *Co inflata* has evolved the capacity to brood self-fertilized embryos in a modified, “inflated” atrial chamber (as reflected in the name of the species; Cohen 1990). Thus, the genomic resources presented herein will facilitate future investigations into the evolutionary mechanisms underlying the gain and loss of self-fertility and associated shifts in morphology. More generally, these resources will help fill gaps in our understanding of the last common tunicate ancestor and the most recent common ancestor of tunicates and vertebrates.

## Materials and Methods

### Reproducibility and Transparency Statement

Custom scripts, command lines, and data used in these analyses and alignment and tree files are available at https://github.com/josephryan/2019-DeBiasse_etal_CorellaGenome. To maximize transparency and minimize confirmation bias, phylogenetic analyses were planned *a priori* in a phylotocol (DeBiasse and Ryan 2019) which was posted to our GitHub repository (URL above).

### DNA Isolation and Genome Sequencing

We extracted genomic DNA from the sperm of a single adult *Co. inflata* (fig. 1) collected at the Roche Harbor repair dock in San Juan Island, WA on August 12, 2013. More details regarding sperm isolation and DNA extraction are available in the Supplementary Material online. We estimated the DNA concentration (208 μg/ml) using a Qubit fluorometer and stored the sample at 4 °C until sequencing. Pacific Biosciences (PacBio) and Illumina DNA libraries were constructed and sequenced at the University of Florida Interdisciplinary Center for Biotechnology Research. PacBio libraries were sequenced on five RS2 SMRT cells and Illumina 100-bp paired-end libraries with 550-bp inserts were sequenced on a HiSeq-2500.

### Genome Assembly

We ran Trimmomatic v0.36 (Bolger et al. 2014) as implemented in the Galaxy server (Afgan et al. 2016) to remove adaptor sequences from the Illumina reads with a sliding window of 4 and an average Phred quality score cutoff of 27. We used Jellyfish v2.2.3 (Marçais and Kingsford 2011) to count k-mers in the Illumina reads and then used Quake v0.3 (Kelley et al. 2010) to correct substitution sequencing errors. We assembled trimmed and error-corrected Illumina reads into contigs using Meraculous v2.2.2.4 (Chapman et al. 2011). We generated artificial mate pairs of size 2, 5, 10, and 15 kb from our PacBio reads using matemaker v1.0 (github.com/josephryan/matemaker). We then scaffolded the Illumina contigs with these mate pairs using SSPACE_Standard v3.0 (Boetzer et al. 2011).

### RNA Isolation and Transcriptome Sequencing

We collected 15 *Co. inflata* individuals on Friday Harbor, WA, on August 8–15, 2015, brought them back to Friday Harbor Lab, and allowed them to spawn in a sea-table. We pooled a wide range of embryonic stages along with hatched larvae in Eppendorf tubes, pipetted vigorously to remove follicle cells, allowed the embryos and larvae to settle, and then rinsed them in 500 μl of 0.2-μm filtered seawater. The tubes were spun down at 3,000 rpm for 1 min, excess water was removed, and samples were frozen in liquid nitrogen and stored at −80 °C until RNA isolation. All samples were pooled and total RNA was isolated using the Qiagen RNeasy Lipid Tissue Mini Kit and treated with DNAase. We checked RNA quality on an Agilent bioAnalyzer chip and sent the RNA to the University of Pennsylvania Next Generation Sequencing Core, where a library was generated using Illumina TruSeq Stranded Total RNA with Ribo Zero Gold. This library was sequenced using an Illumina HiSeq 2500 to generate 100-bp paired-end reads.

### Reference Transcriptome Assembly

We trimmed adaptors from the *Co. inflata* RNA-Seq reads with the Agalma program bl-filter-illumina v0.4.0 (Dunn et al. 2013) and assembled a transcriptome in Trinity v2.4.0 (Haas et al. 2013). We aligned reads to the Trinity assembly with the program align_and_estimate_abundance.pl from the Trinity package and created a new assembly keeping only the isoforms with the highest number of aligned reads using the script rsemgetbestseqs.py (bitbucket.org/wrf/sequences/src). We collapsed contigs in CDHIT v4.7 (Fu et al. 2012) using a 97% similarity threshold and translated the nucleotide transcriptome sequences into amino acid sequences in TransDecoder v5.0.2 (github.com/TransDecoder). We set the TransDecoder “-m” flag to 50 and used the results from BLASTP (McGinnis and Madden 2004) and hmmscan (Johnson et al. 2010) searches to inform the final TransDecoder prediction step.

### Gene Prediction

We inferred gene models for *Co. inflata* in Augustus v3.2.3 (Stanke et al. 2006). First, we created hints by aligning our assembled transcriptome to our genome assembly using BLAT v35x1 (Kent 2002), filtering these alignments with the Augustus utility script filter PSL.pl and then sorting the alignments. We next applied the Augustus utility scripts aln2wig, wig2hints.pl, and blat2hints.pl to create the final hints file for Augustus. In the final prediction step, we set the *Ciona* training set as the value for the -species parameter.

### Assembly Completeness

We assessed the completeness of the *Co. inflata* transcriptome, gene models, and genome by searching against the eukaryote database in BUSCO v2 (Simão et al. 2015) and CEGMA v2.5 (Parra et al. 2007) as implemented in gVolante v1.2.0 (Nishimura et al. 2017).

### Orthogroup Identification and Phylogeny Estimation

We used OrthoFinder v2.2.3 (Emms and Kelly 2015) to identify orthologous groups of sequences in 37 tunicate and 10 outgroup taxa (supplementary table S1, Supplementary Material online). First, we translated the *Co. inflata* nucleotide transcriptome generated in this study and 18 previously published nucleotide transcriptomes into amino acid sequences in TransDecoder v5.0.2 (github.com/TransDecoder). This included 16 transcriptomes from Alié et al. (2018) and 2 from Delsuc et al. (2018); the 18 tunicate and 10 outgroup sequences from Kocot et al. provided to us directly by the authors were already translated. We set the –m flag to 50 and used the results from BLASTP and hmmscan searches to inform the final TransDecoder prediction step. Next, we used diamond v0.9.22.123 (Buchfink et al. 2015) to perform reciprocal BLASTP searches on all 47 amino acid data sets and generated FASTA files of orthologous sequences in OrthoFinder.

To generate a data set with which to estimate a tunicate phylogeny, we filtered the orthogroups inferred by OrthoFinder as follows. First, we aligned sequences within each orthogroup using MAFFT v7.309Katoh and Standley 2013), trimmed poorly aligned regions with Gblocks v0.91b (Talavera and Castresana 2007) using dynamic parameters generated by Gblockswrapper v0.03, and estimated an ML tree using the multicore version of IQ-TREE v1.5.5 (Nguyen et al. 2015). Next, we retained only the orthogroup trees that had at least 85% of the total taxa (40 out of 47 species) and no more than three species with paraphyletic duplicates (monophyletic duplicates were allowed). We used PhyloTreePruner v1.0 (Kocot et al. 2013) to remove all but one sequence in taxa with monophyletic duplicates (e.g., paralogs), which produced a set of orthologous loci with one sequence per species in at least 85% of our taxa.

We used fasta2phylomatrix (github.com/josephryan/fasta2phylomatrix) to concatenate all of the FASTA-formatted ortholog alignments. We estimated a Bayesian species phylogeny in PhyloBayes v4.1b (Lartillot et al. 2009). We launched two PhyloBayes chains for each of nine random starting trees estimated in the multicore version of IQ-TREE v1.5.5 and one neighbor-joining starting tree also estimated in IQ-TREE. After 6 weeks of runtime, the chains for only one of the runs had converged (i.e., the discrepancy observed across all bipartitions was <0.1). We estimated a consensus tree from the converged run by sampling every 10th tree after a 100 tree burn-in. We also estimated an ML phylogeny in IQ-TREE v1.5.5. Models of amino acid substitution for each gene partition were selected by IQ-TREE v1.5.5 using the “-m TEST” parameter. Support values were determined from 1,000 bootstrap replicates. The Bayesian topology differed from the ML topology for one clade (see Results). To compare these alternative topologies, in IQ-TREE v1.5.5, we estimated likelihood score for the data constrained to the Bayesian topology and then compared the likelihood score to our unconstrained ML tree.

### Testing for Compositional Heterogeneity

**Fig. 2.**
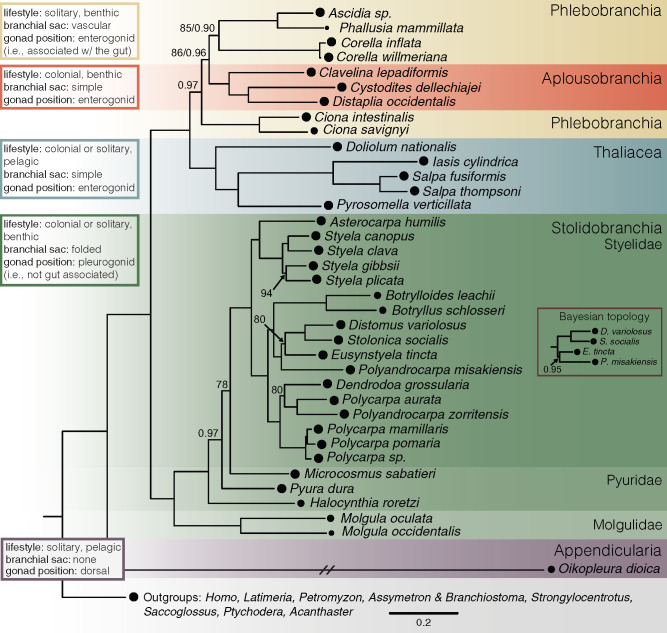
—Tunicate phylogeny. Maximum-likelihood phylogeny of tunicates estimated from a concatenated matrix of 210 orthologous loci identified in transcriptome sequences. Colors represent different levels of taxonomic organization. Circles at the tips represent the occupancy of that taxon in the data matrix. The inset labeled “Bayesian topology” represents the difference between the ML and Bayesian topologies. Nodes with bootstrap values <95 and/or posterior probability values <0.98 are labeled. The branch leading to *Oikopleura dioica* was shortened to fit the figure dimensions. The *Corella inflata* transcriptome was generated in this study. Transcriptomes for other taxa were from Kocot et al. (2018), Alié et al. (2018), and Delsuc et al. (2018). (See supplementary table S1, Supplementary Material online, for full details.) Alignment and tree files are available at https://github.com/josephryan/2019-DeBiasse_etal_CorellaGenome.

**Fig. 3.**
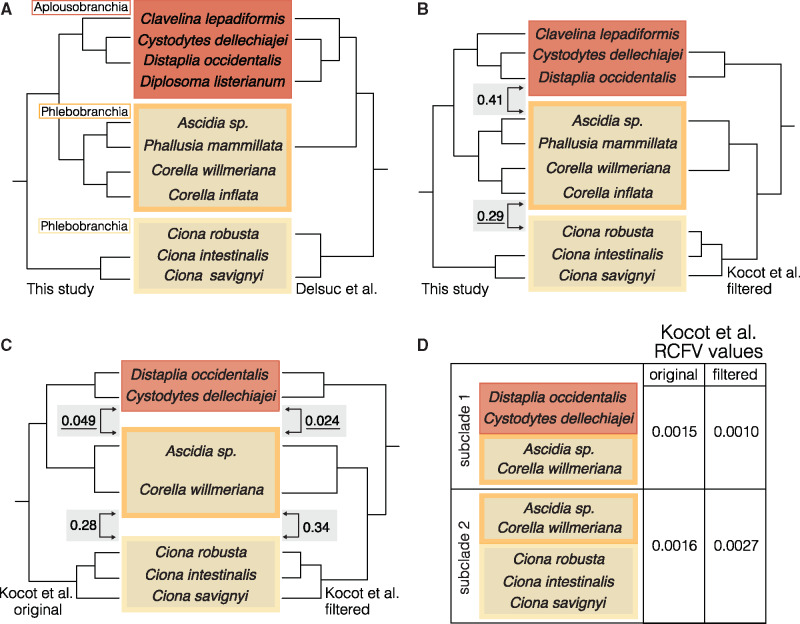
—Alternative topologies and measures of compositional heterogeneity. Yellow shading indicates taxa in Phlebobranchia and red shading indicates taxa in Aplousobranchia. (*A*) Phylogenetic relationships inferred in this study (left) are congruent with those inferred in Delsuc et al. 2018 (right). (*B*) Phylogenetic relationships inferred in this study (left) conflict with those inferred in Kocot et al. 2018 (right). The numbers in gray boxes are chet index values calculated by comparing amino acid compositions of the clades indicated by the arrows. The underlined chet indices specify which clades have more similar amino acid frequencies, which therefore would be expected to be drawn together due to compositional heterogeneity. (*C*) Alternative phylogenetic relationships inferred in Kocot et al. (2018) for the original 798-gene data set (left) and RCFV 50-gene filtered data set (right). The numbers in gray boxes are chet indices of the clades indicated by arrows. (*D*) RCFV values calculated for alternative subclade definitions for the Kocot et al. (2018) original 798-gene data set and RCFV 50-gene filtered data set.

Kocot et al. (2018) used ML and Bayesian inference to estimate a tunicate phylogeny based on a 798-gene concatenated data set and found that Aplousobranchia was nested within a paraphyletic Phlebobranchia: a clade containing *Distaplia occidentalis* and *Cystodites dellechiajei* was sister to a clade containing *Ascidia* sp*.* and *Corella willmeriana*. Kocot et al. (2018) concluded this relationship was caused by compositional heterogeneity, the nonstationarity of nucleotide or amino acid frequencies across a tree (Rodríguez-Ezpeleta et al. 2007). Therefore, they used BaCoCa 1.104.r (Kück and Struck 2014) to calculate the average relative compositional frequency variability (RCFV) score for each gene based on per-taxon RCFV scores calculated, assigning taxa to the following subclades: Ambulacraria (Hemichordata + Echinodermata), Vertebrata, Cephalochordata, and Tunicata. When Kocot et al*.* (2018) re-estimated the ML phylogeny using a data set containing the 50 genes with the lowest RCFV scores, Phlebobranchia was monophyletic. Our 210-gene concatenated ML and Bayesian phylogenies recovered Aplousobranchia nested within a paraphyletic Phlebobranchia (see Results, figs. 2 and 3*A* and *B*; supplementary fig. S1, Supplementary Material online). Therefore, we tested our gene matrix for compositional heterogeneity using chet v0.03 (github.com/josephryan/chet), a program that produces an index representing the level of compositional heterogeneity (chet index) between two clades. The index is the sum of differences between the amino acid composition of the sequences in each clade. We calculated the chet index for the following comparisons in our data set (fig. 3*B*): 1) the Aplousobranchia clade (*Clavelina lepadiformis*, (*Cy. dellechiajei*, *D. occidentalis*)) versus the *Corella-*Phlebobranchia clade ((*Ascidia* sp., *P. mammillata*),(*Co. inflata*, *Co. willmeriana*)) and 2) the *Corella-*Phlebobranchia clade versus the *Ciona-*Phlebobranchia clade (*Ciona savignyi*, *Ci. intestinalis*). If compositional heterogeneity is causing the Aplousobranchia clade to group with the *Corella*-containing Phlebobranchia clade, it is expected that the chet index for comparison 1 will be lower than for comparison 2. We also tested the 798-gene original full data set and 50-gene RCVF data set from Kocot et al. (2018) with chet for the following comparisons (fig. 3*C*): 3) the Aplousobranchia clade (*Cy. dellechiajei, D. occidentalis*) versus the *Corella-*Phlebobranchia clade (*Ascidia* sp., *Co. willmeriana*) and 4) the *Corella-*Phlebobranchia clade versus the *Ciona-*Phlebobranchia clade ((*Ci. savignyi*), (*Ci. robusta*, *Ci. intestinalis*)). Finally, we used BaCoCa v1.105.r to calculate RCFV scores for the original 798-gene and RCFV 50-gene filtered Kocot et al. (2018) data sets, differing from the BaCoCa analyses from the original study by assigning taxa into the following subclades: (1-paraphyletic Phlebobranchia) *Cy. dellechiajei*, *D. occidentalis*, *Ascidia* sp., *Co. willmeriana* and (2-monophyletic Phlebobranchia) *Ascidia* sp., *Co. willmeriana*, *Ci. robusta*, *Ci. intestinalis*, *Ci. savignyi* (fig. 3*D*).

### Hox Gene Analyses

We used hmm2aln.pl (github.com/josephryan/hmm2aln.pl) with the homeodomain hidden Markov model (hd60.hmm) from Zwarycz et al. (2016) to generate an alignment of putative homeodomains from the *Co. inflata*-translated transcriptome and translated gene models and from the *Ci. robusta-*translated transcriptome and translated gene models. To this alignment, we added HOXL subclass homeodomain sequences for *Branchiostoma floridae* from the homeodomain database HomeoDB (Zhong and Holland 2011), and estimated an ML tree using the multicore version of IQ-TREE v1.5.5. Next, we used the program make_subaligment v0.05 (github.com/josephryan/make_subalignment) to prune non-Hox/ParaHox homeodomains from our data set, retaining all sequences from the smallest clade that included the entire set of *B. floridae* Hox and ParaHox sequences. We then estimated an ML gene tree for this alignment in IQ-TREE v1.5.5.

Our preliminary tree contained *Co. inflata* and *Ci. robusta* homeodomains from translated gene models for *Hox1*, *Hox3*, *Hox4*, *Hox10*, *Hox12*, and Cdx (supplementary fig. S2, Supplementary Material online). *Hox2*, *Hox5*, *Hox13*, and Gsx were only represented in *Co. inflata* by a transcript, so we manually created gene models for these Hox genes after confirming that they were in the genome, and then added them to our alignment. Xlox/Pdx was not present in our *Co. inflata* transcriptome or gene models, but was present in the genome, so we manually created a gene model and added it to the alignment. Our method failed to identify a gene model or transcript for *Ci. robusta* *Hox6* (supplementary fig. S2, Supplementary Material online); therefore, we added the *Ci. robusta* *Hox6*/*A7*/*A8* sequence from Aniseed (gene id: Cirobu.g00016147) to our alignment. Our tree included a *Co. inflata* transcript and *Ci. robusta* gene model which were sister to each other on a long branch (supplementary fig. S2, Supplementary Material online). We identified these as engrailed homeodomains, which are considered members of the NKL subclass and are often associated with Hox genes (Holland et al. 1997), and removed them from the alignment. Next, we reran our ML analysis using only homeodomains from gene models, removing any duplicates due to gene model isoforms.

In the final tree, several tunicate Hox genes did not form clades with the *B. floridae* genes of the same name (see Results and supplementary fig. S3, Supplementary Material online). We used an approximately unbiased (AU) test (Shimodaira 2002) implemented in IQ-TREE v1.5.5 to determine whether constraint trees requiring tunicate Hox genes to cluster with the corresponding *B. floridae* Hox loci were significantly different than the unconstrained maximum-likelihood Hox gene tree (supplementary table S2, Supplementary Material online).

To compare the Hox gene complement and genomic orientation of Hox clusters across tunicate taxa and to test the effect of outgroup sequences, we conducted an expanded phylogenetic analysis of Hox genes across seven tunicate species and five outgroup species. First, we searched the genomes of *Ci. savignyi* (Vinson 2005), *Botrylloides leachii* (Blanchoud et al. 2018), *H. roretzi* (Sekigami et al. 2017), *O. dioica* (Seo 2001), and *Molgula oculata* (https://www.aniseed.cnrs.fr) with TBLASTN using the *B. floridae* Hox gene protein sequences as the query and recorded the scaffold number and homeodomain coordinates of each homeobox within each species (supplementary table S3, Supplementary Material online). We aligned the corresponding homeodomains with those identified in *Co. inflata*, *Ci. robusta*, *B. floridae* as described above, and estimated an ML tree using the multicore version of IQ-TREE v1.5.5.

Finally, we determined patterns of Hox gene linkage (i.e., identification of physical linkages on the same chromosome) in *Co. inflata*. Due to the draft nature of the *Co. inflata* genome, the homeoboxes of some Hox genes, those that contained introns, spanned multiple genomic scaffolds in *Co. inflata* (supplementary fig. S5 and table S3, Supplementary Material online). Additionally, some Hox genes that were linked in *Ci. robusta* (Satou et al. 2019) were not linked in our *Co. inflata* genome assembly. We attempted to bridge these gaps with PCR. We designed PCR primers based on the PacBio sequences to link 1) *Hox2* to *Hox4*, 2) *Hox3* to *Hox4*, and 3) *Hox5* to *Hox6*. We amplified genomic DNA (isolated as described above) in 50 µl reactions with Platinum Hi-Fi Taq polymerase (Thermo Fisher) and ran the PCR product on 1% agarose gels to determine the size of the amplicons. To compare patterns of linkage in *Co. inflata* to other tunicates, we used BLAST to find the genome scaffold and coordinate information for the Hox genes and searched previously published studies to determine if Hox genes on different scaffolds had been joined by other methods (e.g., PCR, FISH).

### Gene Loss Analyses

Tunicates are thought to have undergone extensive gene loss since diverging from the last common chordate ancestor (Dehal 2002; Hughes and Friedman 2005; Berná and Alvarez-Valin 2014). Therefore, we searched for gene loss in key developmental gene families TGF-beta, DKK, and FGF and in genes associated with cardiovascular and endothelial lineages (Bhasin et al. 2010) using hidden Markov models and phylogenetic approaches. For the TGF-beta gene family, we used hmm2aln.pl with a hidden Markov model downloaded from Pfam (PF00019) to generate an alignment of putative TGF-beta family genes from the *Co. inflata*-translated transcriptome and translated gene models and from the *Ci. robusta-*translated transcriptome and translated gene models. To this alignment, we added *Homo sapiens* TGF-beta family genes sequences and estimated an ML tree in IQ-TREE v1.5.5. For instances where there were multiple tunicate sequences for one TGF-beta family gene, we blasted the transcripts and/or gene model against the appropriate tunicate genome and removed one duplicate from the pair if both hit the same genomic region. For the smaller gene families, we used the human protein sequences for each gene category as a query to search the *Ci. robusta* and *Co. inflata* protein gene model and translated transcriptome sequences using BLASTP. We retained the top ten tunicate BLAST matches and used BLASTP to search these putative tunicate candidate genes against the Human Reference Sequence (RefSeq) protein gene models. We retained the tunicate candidate genes that were reciprocal best BLAST hits to target human genes. We aligned the tunicate sequences with the corresponding human sequences in MAFFT v7.309, and estimated a gene tree for each gene family in IQ-TREE v1.5.5.

## Results

### Genome Sequencing, Assembly, and Gene Models

We generated 182,320,177 Illumina genomic DNA reads (100 bp paired ended) and 754,194 PacBio genomic DNA reads with an average length of 3,441 bp. We assembled these data into 134,182 scaffolds consisting of 131,290,315 bp with an N50 of 7,263 (supplementary table S4, Supplementary Material online). BUSCO scores for complete core eukaryotic genes and complete plus partial core genes were 245 (81%) and 280 (92%), respectively. CEGMA scores were 197 (79%) for complete core genes and 236 (95%) for complete plus partial genes. The BUSCO scores for the *Co. inflata* gene models were 192 (63%) for complete genes and 247 (82%) for complete plus partial genes (supplementary table S4, Supplementary Material online). Although this *Co. inflata* draft genome assembly is suboptimal compared with other published tunicate genomes (supplementary table S5, Supplementary Material online), it is sufficient to answer the questions about tunicate phylogeny and gene family evolution that we address herein.

### Transcriptome Sequencing and Assembly

We assembled 1,217,050,408 Illumina RNA-Seq reads from *Co. inflata* embryos of a wide range of stages into 147,142 transcripts with a total length of 151,076,728 bp and an N50 of 2,071. We identified 293 (97%) complete genes and 299 (99%) complete plus partial genes. There were 1.83 orthologs per core gene and the GC content was 38%. We translated this transcriptome assembly using TransDecoder into 131,794 protein sequences with a total length of 27,907,540 amino acids. These translations had high BUSCO scores with 293 (97%) complete genes and 300 (99%) complete plus partial core eukaryotic genes present (supplementary table S4, Supplementary Material online).

### Tunicate Gene Matrix and Phylogeny

We generated orthogroups from the 37 translated tunicate and 10 outgroup transcriptomes. We assigned 1,442,493 of 1,782,182 genes (81%) to 49,979 orthogroups. From these orthogroups, we recovered 1,330 orthogroups with at least 40 of 47 species (tunicates + outgroups) present and no more than eight duplicates per species. We removed duplicates that represented likely paralogs or isoforms, yielding 210 single-copy orthogroups.

We constructed a concatenated matrix containing 54,788 amino acid columns and an overall occupancy of 91% (each partition included at least 31 tunicates). All but six nodes in the resulting ML tree were assigned bootstrap values of 100 (fig. 2). Only one of the ten paired Bayesian analyses converged (maxdiff = 0.0165289, 687 total trees) after 6 weeks (running on eight processors each). We estimated the majority-rule posterior consensus tree for these chains (supplementary fig. S1, Supplementary Material online). We found that the converged Bayesian topology and the ML topology were concordant with one exception: in the Bayesian tree, *Eusynstyela tincta* and *Polyandrocarpa misakiensis* were monophyletic and sister to a clade containing *Distomus variolosus* and *Stolonica socialis* (supplementary fig. S1, Supplementary Material online), whereas in the ML tree, *Po. misakiensis* was sister to a clade containing *E. tincta*, which itself was sister to the clade containing *Disto. variolosus* and *S. socialis* (fig. 2).

To choose between differing topologies, we decided *a priori* (in our phylotocol) to compare the two phylogenies using likelihood criteria. We generated an ML tree using the Bayesian topology as a constraint. The likelihood score for the best ML topology (−1,800,144.048) was higher than the likelihood score tree constrained to the Bayesian topology (−1,800,166.082). Therefore, we report the ML topology in the main text (fig. 2) with bootstrap and posterior probability support values at the nodes. The Bayesian topology is reported in supplementary figure S1, Supplementary Material online. Differences in these topologies had no bearing on our main findings.

### Comparison with Previous Phylogenies

The phylogenetic relationships in our species tree largely corroborate previous phylogenomic studies, some of which have revealed discrepancies between phylogeny and taxonomy. For example, as in our study (fig. 2 and supplementary fig. S1, Supplementary Material online), Alié et al. (2018) and Delsuc et al. (2018) tested relationships within Stolidobranchia and found the family Pyuridae to be paraphyletic. Alié et al. (2018) included several *Polycarpa* and *Polyandrocarpa* species and found both genera to be paraphyletic, as did we (fig. 2 and supplementary fig. S1, Supplementary Material online). Another major conflict between phylogeny and taxonomy regards the monophyly of Phlebobranchia. In both our ML and Bayesian topologies, the order Aplousobranchia was nested within a paraphyletic Phlebobranchia (fig. 2 and supplementary fig. S1, Supplementary Material online), a result that corroborates the results shown by Delsuc et al. (2018) (fig. 3*A*) and the majority of the trees (19/25) estimated by Kocot et al. (2018). However, Kocot et al. (2018) hypothesized paraphyly in Phlebobranchia was due to systematic error caused by compositional heterogeneity and recovered a monophyletic Phlebobranchia when re-estimating the phylogeny with a 50-gene data set filtered to reduce compositional heterogeneity. This result motivated us to test whether phlebobranchid paraphyly in our phylogeny was also an artifact caused by compositional heterogeneity.

### Phlebobranchia and Compositional Heterogeneity

Compositional heterogeneity, the nonstationarity of nucleotide or amino acid frequencies across taxa in a tree, can cause unrelated taxa with similar frequencies to group together, and could explain why recent tunicate phylogenies have recovered Phlebobranchia as paraphyletic. Our comparison of the Aplousobranchia clade and the *Corella-*Phlebobranchia clade for our 210-gene data set produced a chet index of 0.41, whereas the chet index comparing the *Ciona*-Phlebobranchia clade to the *Corella-*Phlebobranchia clade was 0.29 (fig. 3*B*). These results indicate that amino acid frequencies are more similar (i.e., the scores are lower) between the *Corella-*Phlebobranchia clade and the *Ciona-*Phlebobranchia clade than between the Aplousobranchia and the *Corella-*Phlebobranchia. These results do not support the hypothesis that compositional heterogeneity caused Aplousobranchia and the *Corella* phlebobranchids to form a clade, making Phlebobranchia paraphyletic.

We applied the chet index to the original 798-gene and the 50-gene RCVF-filtered data sets (hereafter original and filtered) from Kocot et al. (2018). For the original data set, we found that the chet index for the Aplousobranchia and *Corella*-Phlebobranchia clades was 0.049, whereas the index for the *Corella*-Phlebobranchia and *Ciona-*Phlebobranchia clades was 0.28 (fig. 3*C*). For the filtered data set, we found that the chet index for the Aplousobranchia and *Corella*-Phlebobranchia clades was 0.034, whereas the index for the *Corella*-Phlebobranchia and *Ciona-*Phlebobranchia clades was 0.28 (fig. 3*C*). The results for the original Kocot et al. (2018) data set are congruent with the hypothesis that compositional heterogeneity caused Aplousobranchia and the *Corella* phlebobranchids to form a clade, making Phlebobranchia paraphyletic. However, according to the chet indices, filtering made the amino acid frequencies between Aplousobranchia and the *Corella* phlebobranchids more similar (i.e., the score decreased) and the amino acid frequencies between the *Corella* phlebobranchids and the *Ciona* phlebobranchids less similar (i.e., the score increased) (fig. 3*C*). These results suggest the change in topology and subsequent restoration of monophlyly in Phlebobranchia is not due to reduced compositional heterogeneity in the filtered 50-gene data set compared with the original data set.

To further test for compositional heterogeneity, we calculated RCFV scores for the original 798-gene and RCFV 50-gene filtered Kocot et al. (2018) data sets in BaCoCa, assigning taxa into the following: subclade-1: paraphyletic Phlebobranchia (i.e., *Cy. dellechiajei*, *D. occidentalis*, *Ascidia* sp., *Co. willmeriana*) and subclade-2: monophyletic Phlebobranchia (i.e., *Ascidia* sp., *Co. willmeriana*, *Ci. robusta*, *Ci. intestinalis*, *Ci. savignyi*; fig. 3*D*). In the original data set, the RCFV score was 0.0015 for subclade-1 and was 0.0016 for subclade-2. In the filtered data set, the RCFV score was 0.001 for subclade-1 was and was 0.0027 for subclade-2. Based on how we defined the tunicate subclades, the RCFV scores for the original Kocot et al. (2018) data set are congruent with the hypothesis that compositional heterogeneity caused Aplousobranchia and the *Corella* phlebobranchids to form a clade, making Phlebobranchia paraphyletic. However, compositional heterogeneity increased (i.e., the RCVF score increased) for the Phlebobranchia subclade and decreased (i.e., the RCVF score decreased) for the Phlebobranchia and Aplousobranchia subclade (fig. 3*D*). These results suggest that filtering the data set actually increased compositional heterogeneity compared with the original data set for these taxa.

### Relationships within Thaliacea

Relationships of the major lineages within Thaliacea remain controversial. Transcriptomic data from Doliolida, Salpida, and Pyrosomatida were generated as part of the aforementioned phylogenomic studies, but none of these studies analyzed all three of these taxa together. Here we include representatives from all three major Thaliacea lineages. We recovered Doliolida as sister to a clade that included Salpida and Pyrosomatida. The thaliacean relationships in our analyses are congruent with those of the 18S tree in Tsagkogeorga et al. (2009) but conflict with the 18S tree in Govindarajan et al. (2011) and the 18S plus morphological trait-based tree in Braun et al. (2020).

### Hox Gene Analyses

**Fig. 4.**
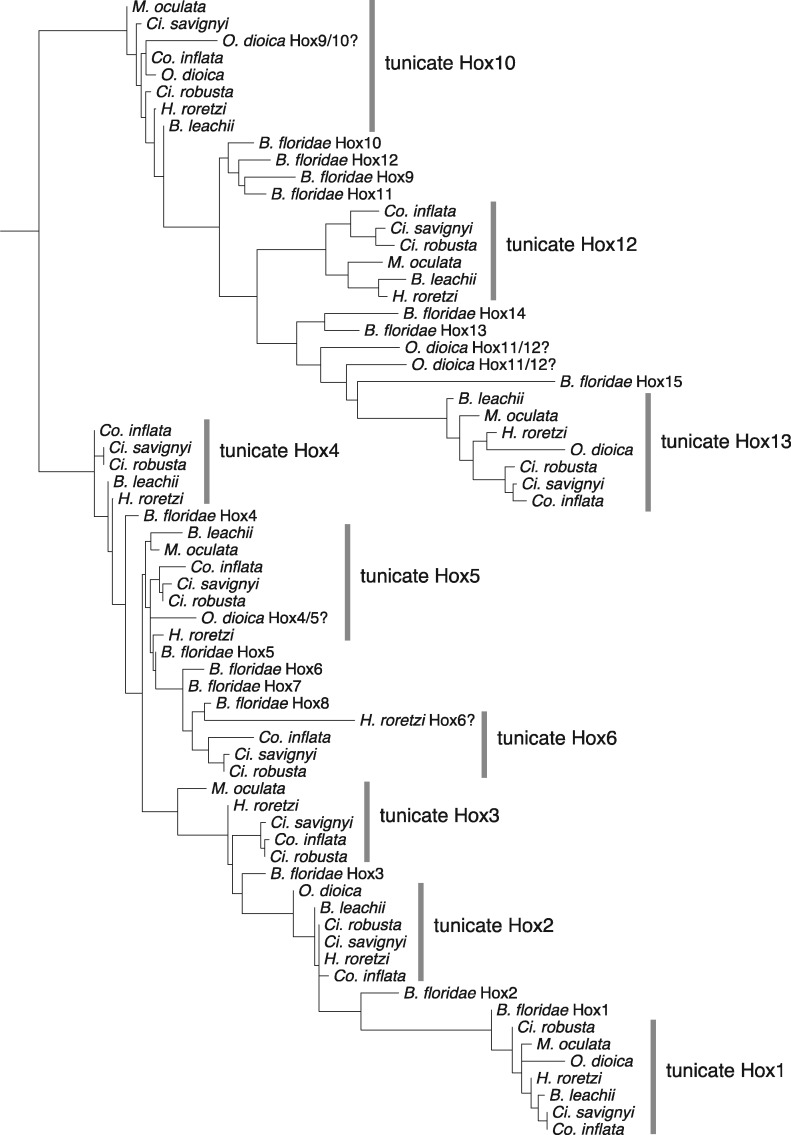
—Tunicate Hox phylogeny. Maximum-likelihood phylogeny of Hox gene homeodomain sequences for *Branchiostoma floridae* and the following tunicate species: *Ciona savignyi*, *Halocynthia roretzi*, *Molgula oculata*, *Botrylloides leachii, Corella inflata*, and *Ciona robusta*. The tree is rooted at the midpoint. Alignment and tree files are available at https://github.com/josephryan/2019-DeBiasse_etal_CorellaGenome.

**Fig. 5.**
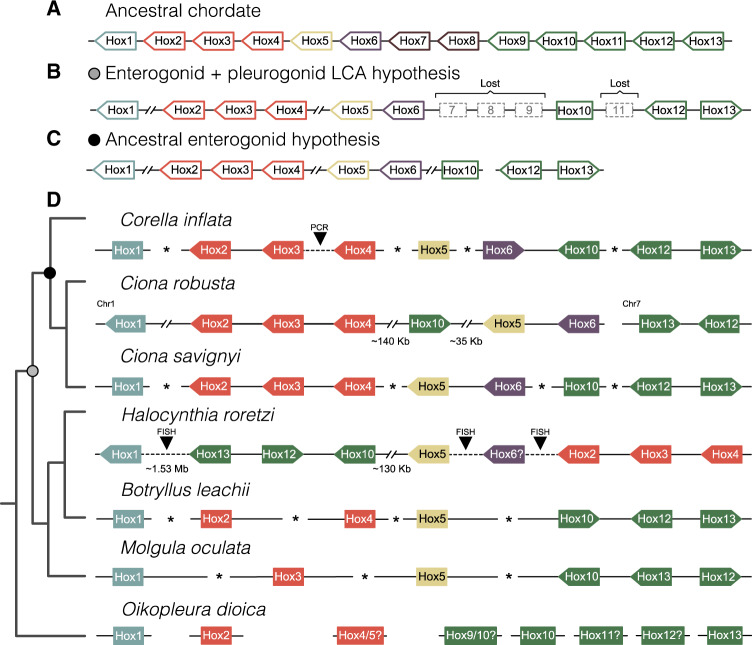
—Genomic organization of Hox genes in tunicates and the chordate ancestor. Linked Hox genes are connected by solid lines. Dashed lines indicate Hox genes that are currently located on separate genomic scaffolds but were shown to be linked using other methods (e.g., FISH, PCR). Asterisks between Hox genes indicate that linkage is unknown. The distances between Hox genes are not to scale. Distances of at least 35 kb are indicated with paired forward slashes. If known, the transcription direction for linked genes is indicated by the direction of the arrow. Non-Hox genes that may be present between Hox genes are not shown. Chromosome numbers and linkage information for *Ciona robusta* are from Satou et al. (2019). (*A*) Hox cluster in the ancestral chordate. (*B*) Inferred Hox cluster in the last common ancestor of enterogonid and enterogonid tunicates. The gray circle represents the position of this ancestral in the tunicate tree. (*C*) Inferred Hox cluster in the enterogonid ancestor. The black circle represents the position of the ancestral enterogonid in the tunicate tree. (*D*) Linkage information for extant tunicates. The linkage shown here for *Ci. robusta* is notably different from that in Blanchoud et al. (2018) who did not report the FISH results from Ikuta et al. (2004). The cladogram on the left shows the evolutionary relationships between taxa. Scaffold identification numbers and sequence coordinates for tunicate Hox genes are available in supplementary table S3, Supplementary Material online.

We reassigned three Hox genes in *H. roretzi* based on their relationship to *Ci. robusta* and other tunicate Hox genes (figs. 4 and 5;supplementary fig. S4 and table S3, Supplementary Material online): *Hox6* (previously named *HoxX*), *Hox12* (previously named *Hox11/12/13a*), and Hox13 (previously named *Hox 11/12/13 b*; Sekigami et al. 2017). We also reassigned three Hox genes in *M. oculata* (figs. 4 and 5; supplementary table S3, Supplementary Material online): *Hox10* (originally identified as *Hox12*), *Hox12* (originally identified as *Hox10*), and *Hox13* (originally identified as *Hox11*; Blanchoud et al. 2018). The phylogenetic placement of *O. dioica Hox4*, *Hox9*, *Hox11*, and *Hox12* is ambiguous (figs. 4 and 5; supplementary fig. S4 and table S3, Supplementary Material online), but we retain the current classifications. We found that *Co. inflata* has the same set of Hox genes as *Ci. robusta*, *Ci. savignyi*, and *H. roretzi* (*Hox1-6*, *Hox10*, *Hox12-13*) (figs. 4 and 5;supplementary fig. S4 and table S3, Supplementary Material online).

Several previously named tunicate Hox clades failed to form a monophyletic group with the correspondingly named *B. floridae* Hox genes. However, our AU testing demonstrated that trees constrained to produce relationships consistent with naming were not significantly worse than unconstrained trees (supplementary table S2, Supplementary Material online). Therefore, in *Co. inflata*, we classify *Hox4*, *Hox5*, *Hox6*, and the posterior Hox genes *Hox10*, *Hox12*, and *Hox13* based on the historical naming of these genes in *Ci. robusta*, although we maintain that their true orthology in relation to other chordates remains ambiguous (see Discussion).

We identified a *Co. inflata* genomic scaffold that included the homeoboxes of *Hox12* and *Hox13* (separated by 7,676 bp) and another genomic scaffold with the homeoboxes of Hox6 and Hox10 (separated by 985 bp; fig. 5*D* and supplementary table S3, Supplementary Material online). We recovered *Co. inflata Hox2*, *Hox3*, and *Hox4* on individual scaffolds. However, using a PCR approach, we showed that *Hox2*, *Hox3*, and *Hox4* homeoboxes are present within the same 60-kb stretch of the *Co. inflata* genome (supplementary fig. S5 and table S3, Supplementary Material online). We made similar PCR-based efforts but failed to link *Hox10* to *Hox5*, or *Hox5* to *Hox6* in *Co. inflata*. We recovered the ParaHox genes *Cdx*, *Gsx*, and *Xlox/Pdx* on individual scaffolds in *Co. inflata*.

### Gene Loss Analyses

**Fig. 6.**
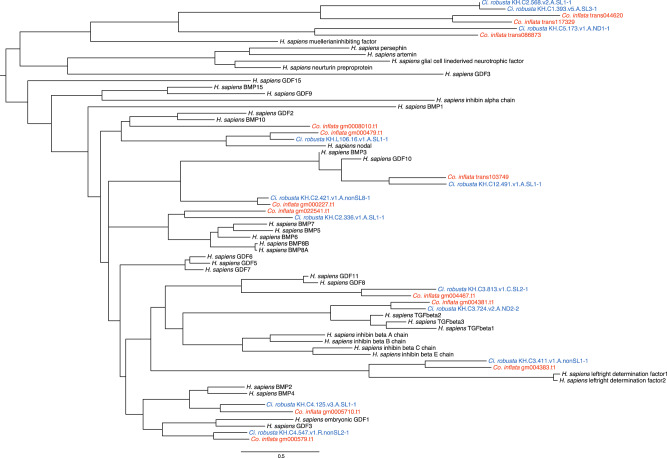
—*TGF-beta* family gene tree. Maximum-likelihood gene tree for *Homo sapiens*, *Ciona robusta*, and *Corella inflata* TGF-beta gene family sequences. Tree is rooted at the midpoint. Alignment and tree files are available at https://github.com/josephryan/2019-DeBiasse_etal_CorellaGenome.

Given that the Ciona lineage is missing some key genes related to cardio-vascular development and function, we surveyed *Ci. robusta* and *Co. inflata* for these gene families. We found that both *Ci. robusta* and *Co. inflata* shared the same complement of DKK genes indicating no losses (supplementary fig. S6, Supplementary Material online). Further, we found that *Ci. robusta* is missing *BMP10*, which is present in *Co. inflata* (fig. 6). In our FGF gene tree, we found that one *Ci. robusta* sequence is missing a *Co. inflata* ortholog (supplementary fig. S7, Supplementary Material online). However, the relationship of the unpaired *Ci. robusta* sequence to a human FGF is ambiguous; although the reciprocal best BLAST hit for this *Ci. robusta* sequence is an FGF gene, the difference between the e-value of the top hit and a non-FGF hit is small, suggesting it may not be a true FGF gene or it may be a highly derived FGF. We also found that *Ci. robusta* appears to have lost the cardiovascular-associated DNA-binding transcription factor *vasculin-like protein-1*. Because *BMP10* is also strongly associated with cardiovascular development, we focused on additional endothelial-associated genes and found two more, a glucose transporter (*SCL2A12*, XP_016865800.1) and a cyclic phosphodiesterase (*PDE2a*, NP_002590) that also appear to be lost in *Ciona*. Finally, we identified an unannotated reading frame in the *Ci. robusta* genome that matched *epicardin*, a cardiovascular-associated transcription factor that we originally thought was absent from *Ci. robusta*. Interestingly, this gene was not predicted and has not been detected in *Ci. robusta* transcriptomes, and thus may represent a pseudogene.

## Discussion

Confidence in phylogenetic relationships and patterns of molecular and phenotypic trait evolution in tunicates is critical to interpreting the extensive experimental developmental biology research in tunicates within an evolutionary framework. The generation of genomic resources for additional species across the tunicate tree also provides insight into how well results for the long-time model *Ci. robusta* represent tunicates as a whole. Toward this goal, we present the genome of *Co. inflata*, an updated tunicate tree of life, analyses of the evolution of the tunicate Hox cluster, and an analysis of gene loss in *Ciona* and *Corella* lineages.

### The State of Tunicate Genomics

To date, there are complete genomes publicly available for 16 tunicate species (supplementary table S5, Supplementary Material online) with an additional four in press (Dardaillon et al. 2019). These genomes will help resolve long-standing questions regarding tunicate evolution and the nature of the ancestral chordate. Here, we report an additional noncionid phlebobranchid genome. This resource is particularly valuable given the importance of cionids to biomedical and evodevo research, especially when considering the genomic variability seen within tunicate clades. For example, the recent sequencing of six additional Appendicularia genomes revealed that genome size varies up to 12-fold across larvaceans (Naville et al. 2019).

In terms of assembly quality, the *Co. inflata* genome is suboptimal to many of the previously published tunicate genomes (supplementary table S5, Supplementary Material online). Nevertheless, we show it to be a useful resource for phylogenomic and gene family analyses. Beyond this work, we have already demonstrated the value of these resources by using them to characterize the evolution of *cis*-regulation in the cardiopharyngeal gene regulatory networks of *Co. inflata* and *Ci. robusta* (Colgan et al. 2019).

### Tunicate Tree of Life

Phylogenetic hypotheses in tunicates have been dynamic over the last 20+ years. Here, we combine transcriptome sequences from three recent tunicate phylogenomic studies (Alié et al. 2018; Delsuc et al. 2018; Kocot et al. 2018) with new data from *Co. inflata*, expanding taxon sampling, and moving us closer to resolving a comprehensive tunicate tree of life.

Historically, tunicates have been divided into three classes (Ascidiacea, Thaliacea, and Appendicularia) associated with a diverse suite of morphological characters and life history traits, such as colonial versus solitary and benthic versus pelagic lifestyles (Berrill 1936). Under this scheme, Ascidiacea are further subdivided into the Phlebobranchia, Aplousobranchia, and Stolidobranchia based on the branchial sac morphology (Lahille 1886, 1890), an organ used to filter food particles from the water column. However, in concordance with previous studies, we found conflict between this historical view (reflected in current taxonomic classification) and molecular phylogenies, which has important implications for how we interpret the evolution of morphology and life history traits in tunicates. We found Ascidiacea to be paraphyletic, a pattern that has been known for some time (Swalla et al. 2000; Stach and Turbeville 2002; Winchell et al. 2002; Zeng and Swalla 2005; Tsagkogeorga et al. 2009), with Thaliacea sister to a clade containing Phlebobranchia and Aplousobranchia. Concordant with the relationships within Thaliacea found by Tsagkogeorga et al. (2009), but in contrast to other phylogenetic studies (Govindarajan et al. 2011; Braun et al. 2020), we found *Doliolum* to be sister to a clade containing *Salpa* and *Pyrosomella*. Understanding these relationships is important for understanding trait evolution (e.g., pelagic vs. benthic life history and morphological and embryological innovations) in this group (Piette and Lemaire 2015). We recovered Aplousobranchia nested within a paraphyletic Phlebobranchia, a pattern found in the phylogeny presented by Delsuc et al. (2018). These results suggest a dynamic evolutionary history of the tunicate branchial sac with thaliaceans coopting it for jet propulsion and aplousobranchs simplifying it for adaptation to a colonial lifestyle.

Unlike branchial sac morphology or life history traits, gonad position, which was historically used by some authors to classify Ascidiacea (Perrier 1898; Garstang 1928), is congruent with the molecular phylogeny inferred in this study. Phlebobranchia, Aplousobranchia, and Thaliacea, which form a clade in our tree, are classified as Enterogona, with gonads closely associated with the gut. Stolidobranchia, which we find to be sister to the Phlebobranchia+Aplousobranchia+Thaliascea clade, is classified as Pleurogona, with gonads not associated with the gut. Our results support the use of gonad position as a reliable taxonomic morphological trait, an observation also noted by Tsagkogeorga et al. (2009). In light of these data, it is worth considering revising higher taxonomic classifications within Tunicata, specifically considering the use of Enterogona and Pleurogona over the currently favored Phlebobranchia and Aplousobranchia.

In phylogenomics, many sources of systematic error can mislead inferences of evolutionary relationships among taxa. For example, differences in amino acid (and nucleotide) composition are well known to influence phylogenetic estimation (Mooers and Holmes 2000; Foster 2004). In theory, under extreme levels of compositional heterogeneity, two unrelated clades with similar amino acid composition will be drawn together in a phylogenetic analysis. Methods for reducing the effects of compositional heterogeneity have been proposed, for example, amino acid recoding (Embley et al. 2003; Hrdy et al. 2004; Martin et al. 2005), but the efficacy of these methods remains untested or has been refuted (Hernandez and Ryan 2019). Nevertheless, it is imperative to prove that compositional heterogeneity is causing phylogenetic error before it can be used as a reason for rejecting a particular phylogenetic tree.

Kocot et al. (2018) suggested that the paraphyly of Phlebobranchia was an artifact due to compositional heterogeneity and in an effort to combat this, the authors divided taxa into subclades (Ambulacraria (Hemichordata + Echinodermata), Vertebrata, Cephalochordata, and Tunicata), measured compositional heterogeneity in each partition in their original 798-gene data set, and re-estimated the tunicate phylogeny with the 50 genes that had the best RCFV score. This filtered data set restored Phlebobranchia monophyly. However, using a subclade definition focused on the Phlebobranchia and Aplousobranchia specifically, we found that for these taxa the Kocot et al. (2018) filtered data set had increased compositional heterogeneity compared with the original data set. Furthermore, using a straightforward measure of amino acid frequency (chet), we showed that although amino acid frequencies were more similar between Aplousobranchia and the *Corella* Phlebobranchia in the original Kocot et al. (2018) data set, filtering the data did not reduce this similarity (fig. 3*C*). Interestingly, the chet results for our data set showed that although amino acid frequencies were more similar between the two Phlebobranchia clades, a characteristic that would suggest the absence of compositional heterogeneity, these two did not form a clade in our analyses (fig. 3*B*). Taken together, these results suggest that the recovery of a monophyletic Phlebobranchia in the Kocot et al. (2018) filtered set is not due to reduced compositional heterogeneity, but rather to an overall reduction in information. We maintain that our tunicate phylogeny and those obtained by Delsuc et al. (2018) and Alié et al. (2018) offer convincing evidence supporting the paraphyly of Phlebobranchia. Finally, these results demonstrate the ongoing challenge of identifying effective strategies for combatting sources of systematic error, such as compositional heterogeneity, in phylogenomics.

### Hox Gene Cluster Evolution

Hox genes play an important role in embryonic development as key loci in the specification of the primary body axis in bilaterian and cnidarian animals (McGinnis and Krumlauf 1992; Finnerty 2003; Carroll 2005; Holland et al. 2007; Ryan et al. 2007). Hox genes often exist in tight clusters along a single chromosome without intervening non-Hox genes and can exhibit spatial and temporal collinearity, wherein the physical position of the genes along the chromosome corresponds to the position and timing of their expression along the body axis of the developing embryo (Lewis 1978; Izpisúa‐Belmonte et al. 1991). Spatial collinearity is largely conserved across bilaterians, with temporal collinearity restricted to vertebrates, cephalochordates (the amphioxus *Branchiostoma*), and some arthropods and annelids (Monteiro and Ferrier 2006). There are competing views about whether temporal collinearity drives spatial collinearity or vice versa and the importance of temporal collinearity in maintaining Hox genes in clusters (Duboule 1992; Monteiro and Ferrier 2006; Gaunt 2018); nevertheless, it is widely accepted that in most animals, Hox collinearity is important for normal embryonic development (Ferrier and Holland 2002). The growing availability of genome data for a broader group of animals has revealed diverse evolution in the Hox gene family, particularly in tunicates. In all tunicate taxa studied to date, Hox clusters have diverged in terms of gene order and chromosomal compactness relative to the ancestral chordate. An extreme example of this trend is displayed by *O. dioica*, in which each Hox gene appears to be located on a different chromosome without any physical linkage (Seo et al. 2004).

In other instances, tunicate Hox genes are still linked but separated by distances as large as ∼1.53 Mb (e.g., in *H. roretzi*, Sekigami et al. 2017). Interestingly, some coordination of Hox gene expression has been conserved in some tunicates, despite the extreme divergence of the Hox cluster (Ikuta et al. 2004; Seo et al. 2004; Nakayama et al. 2016), calling into question the importance of tight clustering for proper embryonic development, at least for tunicates. Furthermore, knockdown experiments in *Ci. robusta* showed that not all Hox genes play a role in larval development (Ikuta et al. 2004).

Reconstructions of ancestral Hox clusters across nodes of the animal tree allow us to better understand Hox gene duplications, losses, and translocations, and how these genomic changes relate to alterations in development. Accurate ancestral reconstructions depend on correctly identifying Hox gene orthologs and paralog across taxa. Unfortunately, Hox gene trees are notoriously difficult to interpret because the homeodomain sequences commonly used to estimate the phylogenies are short and node support is often low (Holland 2013). Previous tunicate Hox gene trees were somewhat limited by the small number of taxa available (Seo et al. 2004; Sekigami et al. 2017). A strength of our study is our inclusion of seven tunicate species that improved the phylogenetic resolution; however, some ambiguities remain. For example, based on our Hox gene tree, it is unclear whether the *O. dioica* Hox cluster contains *Hox9*, as suggested by Seo et al. (2004), or two copies of *Hox10* and the *O. dioica* Hox gene identified as *Hox4* (Seo et al. 2004) clusters with *Hox5* in our phylogeny. There is also ambiguity in the identity of *O. dioica Hox11* and *Hox12* and *H. roretzi Hox6*.

The convention for naming Hox genes also leads to confusion when drawing conclusions about the evolution of this group of genes. Hox genes of the cephalochordate *B. floridae* were named *Hox1* to *Hox15* according to their position along the chromosome, but these names are not necessarily direct orthologs of the vertebrate Hox genes that share the same name (Scott 1993). In particular, the posterior *B. floridae* Hox genes (*Hox10-15*) are fast evolving and have been especially difficult to classify phylogenetically (Ferrier et al. 2000). In our trees, there were multiple instances where tunicate Hox genes that were given names suggesting orthology to vertebrate Hox did not group with the corresponding *B. floridae* Hox gene (e.g., *Ci. robusta* and *Co. inflata Hox13* grouped with *B. floridae Hox15*, fig. 4 and supplementary fig. S3, Supplementary Material online). Using the approximately unbiased test, we determined that trees in which tunicate Hox genes were constrained to a clade with the corresponding *B. floridae* Hox gene (i.e., tunicate *Hox13* forced to cluster with *B. floridae Hox13*) were not significantly different than an unconstrained Hox tree (supplementary table S2, Supplementary Material online). These results reflect the difficulty in identifying Hox gene orthologs and paralogs across taxa.

Using these new data, we reconstructed the Hox cluster for two ancestral tunicate lineages, the last common ancestor of Enterogona and Pleurogona, and the last common ancestor of Enterogona. Based on our results and those of others, we hypothesize that the last common ancestor of Enterogona and Pleurogona lost *Hox7–9* and *Hox11* (fig. 5*B*). Although remaining Hox genes remained linked in this ancestor (i.e., physically connected to each other on the same chromosome), we propose that the genomic distance between *Hox1* and *Hox2–4* as well as between *Hox2–4* and *Hox5* increased considerably (fig. 5*B*).

Based on the conserved position and transcription direction of *Hox5* and *Hox6* in *Ci. robusta*, *Ci. savignyi*, and the ancestral chordate (fig. 5*A* and *D*), the most parsimonious explanation is that this arrangement was present in the ancestral enterogonid (fig. 5*C*) and perhaps lost in *Co. inflata*, in which *Hox5* and *Hox6* appear to be unlinked (fig. 5*D*; although future chromosome-level assemblies may show they are distantly linked). In *Co. inflata*, the tight linkage between *Hox6* and *Hox10*, an arrangement expected after the loss of *Hox7–9* in the stem tunicate, suggests that Hox6 and *Hox10* were tightly linked in the ancestral enterogonid. Together this suggests a tight cluster of *Hox5*, *Hox6*, and *Hox10* in the ancestral enterogonid, and also that the translocation of *Hox10*, which is positioned between *Hox4* and *Hox5* in *Ci. robusta*, occurred after the *Ciona* lineage split from the rest of tunicates. As such, grouping within this *Hox5,6,10* cluster was maintained differentially in descendent enterogonid lineages (e.g., *Hox5–6* in *Ci. robusta* or *Hox6–10* in *Co. inflata*).

Unlike in the enterogonids, *Hox10* is linked to *Hox12* and *Hox13* in *H. roretzi*, *Bo. leachii*, and *M. oculata* suggesting that the tight linkage between these three genes was inherited from the chordate ancestor and was maintained in the lineage leading to the last common pleurogonid ancestor. This contrasts with the enterogonid ancestor where there is currently no evidence linking *Hox12* and *Hox13* to the rest of the Hox cluster.

### Gene Loss

Our analyses showed that orthologs to several important developmental genes present in *Co. inflata* are absent from *Ci. robusta*. This is especially important given the status of *Ci. robusta* as the main experimental tunicate model for evolutionary developmental studies. Strikingly, these lost orthologs include several genes associated with endothelial lineages or more broadly with cardiovascular development including *BMP10*, *vasculin-like protein-1*, a glucose transporter, and a cyclic phosphodiesterase. Further, extensive transcriptomic data indicate that *Ciona epicardin*, another cardiovascular-associated gene, is not expressed, suggesting it may be a pseudogene. These findings may reflect divergent evolutionary shifts in cardiovascular morphology and/or development among different tunicate clades. These findings also suggest that a broad comparative approach will be required to reconstruct the cardiovascular capabilities of the ancestral tunicate as well as the last common ancestor of tunicates and vertebrates.

## Conclusions

Here, we present assembled and annotated genome and transcriptome sequences of the tunicate *Co. inflata*. We have used these data to further resolve controversies in the tunicate tree of life, specifically providing support for the paraphyly of Phlebobranchia, the group that contains *Co. inflata* and the tunicate super model *Ci. robusta*. This phylogeny has implications for the reconstruction of ancestral traits, both phenotypic and genomic. We identify clustered Hox genes, and in light of these data, provide insight into Hox cluster evolution within tunicates. Further, we identify losses of key developmental genes in *Ci. robusta* that have been retained in *Co. inflata*, underlining the importance of establishing additional functional tunicate developmental models. Taken together, these results improve our understanding of development and diversification in tunicates and provide a foundation from which a broad range of functional genomic tools can be applied to test hypotheses about tunicate evolution and the biology of *Co. inflata*.

## Supplementary Material

evaa060_Supplementary_Data

## Notes

**Data deposition:** This project has been deposited at the European Nucleotide Archive under the accession number PRJEB35402. The genome assembly and gene models are available through Aniseed (https://www.aniseed.cnrs.fr) and at http://ryanlab.whitney.ufl.edu/genomes/Core_infl/. Files and scripts required to replicate all analyses are available at https://github.com/josephryan/2019-DeBiasse_etal_CorellaGenome.
