# Supplementary material for: Inferring Tunicate Relationships and the Evolution of the Tunicate Hox Cluster with the Genome of Corella inflata
Source: Genome Biol Evol. 2020 Mar 25;12(6):948–64. doi: 10.1093/gbe/evaa060 (PMC7337526; doi:10.1093/gbe/evaa060)
Supplement: evaa060_Supplementary_Data [file evaa060_supplementary_data.zip › TableS2_AU.docx]

**Table S2 Results of approximately unbiased test to compare alternative hypotheses of tunicate Hox gene phylogenetic relationships**

*________________________________________________________________*______________

Constraint AU test p-value

______________________________________________________________________________

*Co. inflata* Hox4, *Co. robusta* Hox4, *B. floridae* Hox4 0.5353

*Co. inflata* Hox5, *Co. robusta* Hox5, *B. floridae* Hox5 0.7211

*Co. inflata* Hox6, *Co. robusta* Hox6, *B. floridae* Hox6 0.1762

*Co. inflata* Hox10, *Co. robusta* Hox10, *B. floridae* Hox10 0.2090

*Co. inflata* Hox12, *Co. robusta* Hox12, *B. floridae* Hox12 0.4051

*Co. inflata* Hox13, *Co. robusta* Hox13, *B. floridae* Hox13 0.3428

*Co. inflata* Hox10, *Co. robusta* Hox10, 0.1542

*B. floridae* Hox9,10,11,12

*Co. inflata* Hox10,12,13, *Co. robusta* Hox10,12,13, 0.1100

*B. floridae* Hox13,14,15

______________________________________________________________________________
