# Supplementary material for: Inferring Tunicate Relationships and the Evolution of the Tunicate Hox Cluster with the Genome of Corella inflata
Source: Genome Biol Evol. 2020 Mar 25;12(6):948–64. doi: 10.1093/gbe/evaa060 (PMC7337526; doi:10.1093/gbe/evaa060)
Supplement: evaa060_Supplementary_Data [file evaa060_supplementary_data.zip › TableS4_genome_stats.docx]

**Table S4 Genome and transcriptome assembly statistics for *Corella inflata.***

*________________________________________________________________*

Assembly statistics genome transcriptome

________________________________________________________________

Sequence length 131,290,315 151,076,728

Number of scaffolds 134,182 147,142

N50 7263 2071

BUSCO complete 245 (81%) 293 (97%)

BUSCO complete + partial 280 (92%) 299 (99%)

Genes predicted 18,627 NA

________________________________________________________________
