# Supplementary material for: Inferring Tunicate Relationships and the Evolution of the Tunicate Hox Cluster with the Genome of Corella inflata
Source: Genome Biol Evol. 2020 Mar 25;12(6):948–64. doi: 10.1093/gbe/evaa060 (PMC7337526; doi:10.1093/gbe/evaa060)
Supplement: evaa060_Supplementary_Data [file evaa060_supplementary_data.zip › Sup_tables_figures_10March.docx]

*DNA extraction procedure*

Genomic DNA was extracted from the sperm of a single adult C. inflata collected at the Roche Harbor repair dock in San Juan Island, WA on August 12th, 2013. For sperm extraction, the tunic was removed and the body wall dissected away. Fecal matter and intestinal tissue were then removed and the dissected animal was rinsed twice in filtered seawater in a small petri dish. The sperm duct was then torn at the tip and sperm was allowed to pool on bottom of the dish, or was pushed out of the duct. Concentrated sperm was then pipetted into a 2ml Eppendorf tube and placed on ice. Sperm was then resuspended in 1ml filtered seawater by vortexing and spun at 1000 RPM for 2 min to remove large particles (eggs and tissue). Supernatant was transferred to a new tube and spun at 6000 rpm for 2 minute to pellet sperm. Supernatant was removed and purified sperm rinsed twice using the same protocol in 1ml of filtered seawater.

For the second rinse, re-suspended sperm was transferred to a pre-weighed Eppendorf tube so that pelleted sperm weight could be estimated. After weighing, pelleted sperm was frozen in liquid nitrogen and stored until genomic DNA extraction. A sample containing 15μg of purified sperm was used for extraction according to the following protocol:

Sperm was re-suspended in 500μl ice-cold endotoxin-free water and transferred to a fresh 2ml Eppendorf tube (USA). After addition of 500μl of Buffer X2 was then added (20mM TrisHCl pH 8.0, 20mM EDTA, 200mM NaCl, 4% SDS), sperm was mixed very gently by pipetting with a cut P1000 pipette tip to resuspend and incubated at 55°C for 2 hours on a nutator. After cooling back to room temperature, sample was mixed with 1 volume of phenol-chloroform-isoamyl-alchohol (25:24:1) and rotated at room temperature for 10 minutes before being spun down at max speed for 5 min. The aqueous phase was then transferred to a fresh tube and extraction was repeated 3 times so that protein/lipid interphase was no longer visible. At this point two chloroform extractions were performed and the aqueous phase was transferred to a sterile 15ml Corex tube for precipitation (0.1 volume 3M NaOAc, pH 5.2, and 3 volumes ice cold ethanol, mix by inversion). Precipitated DNA was transferred to a fresh 2ml tube using a cut P1000 tip and rinsed with 1ml of 70% ethanol and spun down at max speed for 15 min at 4°C. After removing supernatant, the pellet was allowed to air dry for 10 minutes and re-suspended in 200μl of TE overnight at 4°C in a nutator. To complete re-suspension, gDNA was incubated at 65°C for 15 minutes and mixed gently by pipetting in a cut P1000 tip and stored at 4°C.

A 5μl aliquot was diluted 1:10 in TE and nanodropped to evaluate purity and concentration (230ng/μl, A260/A280 1.88). Diluted sample was also run on an agarose gel to estimate fragmentation, verifying that DNA was not sheared (forming a discrete high molecular weight band well above maximal ladder length). Qubit was then used to precisely estimate gDNA concentration (208 μg/ml) and stored at 4°C until being sent to the University of Florida (UFL) for sequencing. Illumina and PacBio library construction was conducted at UFL. Sequencing was carried out on two separate platforms: Illumina HiSeq-25000 (PE, 100 cycles 100 bp pair-end reads, 550 bp insert) and PacBio 5 SMRT cells RS.

**Figure S1 Bayesian tunicate phylogeny.** Bayesian phylogeny of tunicates estimated from a concatenated matrix of 210 orthologous loci identified in transcriptome sequences. Colors represent different levels of taxonomic organization. Circles at the nodes indicate the level of posterior probabilities at that node according to key. The branch leading to *Oikopleura dioica* was shortened to fit the figure dimensions. The *Corella inflata* transcriptome was generated in this study. Transcriptomes for other taxa were from Kocot et al. (2018), Alié et al. (2018), and Delsuc et al. (2018). See Table S1 for full details. Alignment and tree files are available at https://github.com/josephryan/2019-DeBiasse_etal_CorellaGenome.

**Figure S2 Homeodomain phylogeny.** Maximum-likelihood phylogeny of homeodomain sequences in *Branchiostoma floridae* (black), *Corella inflata* (orange), and *Ciona robusta* (blue). Tunicate transcript sequences are named ‘trans’ followed by the transcript number and tunicate gene models are named ‘gm’ followed by the gene model number. All non-HOXL subclass tunicate sequences are collapsed into one clade. The tree is rooted on *B. floridae Gbx*. Alignment and tree files are available at https://github.com/josephryan/2019-DeBiasse_etal_CorellaGenome.

**Figure S3 Hox/ParaHox phylogeny.** Maximum-likelihood phylogeny of Hox and ParaHox sequences in *Branchiostoma floridae* (black), *Corella inflata* (orange), and *Ciona robusta* (blue). The tree is rooted on *B. floridae Gsx*. Alignment and tree files are available at https://github.com/josephryan/2019-DeBiasse_etal_CorellaGenome.

**Figure S4** Maximum-likelihood phylogeny of Hox sequences in tunicates and outgroup taxa. Alignment and tree files are available at https://github.com/josephryan/2019-DeBiasse_etal_CorellaGenome.

**Figure S5** Stretch of *Corella inflata* genome sequence, inferred using PCR, which includes *Hox2*, *Hox3*, and *Hox4*. Genomic scaffolds from the current *Corella inflata* assembly are dark blue and PacBio sequencing reads aligning to this region are light blue. The homeoboxes of *Hox2*, *Hox3*, and *Hox4* all contain introns (horizontal lines connecting exons represented by vertical lines) and are indicated in dark grey. Arrows above introns indicate the direction. PCR primer pairs used to test physical linkage (green) of the Hox genes are indicated by arrowheads with letters and connected by dashed lines. The scaffold labeled * indicates S14931, the full length of which is not shown. Coordinates for these data are indicated in Table S3.

**Figure S6 DKK gene tree.** Maximum-likelihood gene tree for *Homo* *sapiens*, *Ciona robusta*, and *Corella inflata DKK* sequences. Tree is rooted at the midpoint. Alignment and tree files are available at https://github.com/josephryan/2019-DeBiasse_etal_CorellaGenome.

**Figure S7 FGF gene tree.** Maximum-likelihood gene tree for *Homo* *sapiens*, *Ciona robusta*, and *Corella inflata FGF* sequences. Tree is rooted at the midpoint. Alignment and tree files are available at https://github.com/josephryan/2019-DeBiasse_etal_CorellaGenome.

TABLES

**Table S1 Genome and transcriptome assembly statistics for *Corella inflata.***

*________________________________________________________________*

Assembly statistics genome transcriptome

________________________________________________________________

Sequence length 131,290,315 151,076,728

Number of scaffolds 134,182 147,142

N50 7263 2071

BUSCO complete 245 (81%) 293 (97%)

BUSCO complete + partial 280 (92%) 299 (99%)

Genes predicted 18,627 NA

________________________________________________________________

**Table S4 Results of approximately unbiased tests comparing alternative hypotheses of tunicate Hox gene phylogenetic relationships**

*________________________________________________________________*________

Constraint AU test p-value

________________________________________________________________________

*Co. inflata* Hox4, *Co. robusta* Hox4, *B. floridae* Hox4 0.5353

*Co. inflata* Hox5, *Co. robusta* Hox5, *B. floridae* Hox5 0.7211

*Co. inflata* Hox6, *Co. robusta* Hox6, *B. floridae* Hox6 0.1762

*Co. inflata* Hox10, *Co. robusta* Hox10, *B. floridae* Hox10 0.2090

*Co. inflata* Hox12, *Co. robusta* Hox12, *B. floridae* Hox12 0.4051

*Co. inflata* Hox13, *Co. robusta* Hox13, *B. floridae* Hox13 0.3428

*Co. inflata* Hox10, *Co. robusta* Hox10, 0.1542

*B. floridae* Hox9,10,11,12

*Co. inflata* Hox10,12,13, *Co. robusta* Hox10,12,13, 0.1100

*B. floridae* Hox13,14,15

________________________________________________________________________
